# Increasing influenza vaccination rates via low cost messaging interventions

**DOI:** 10.1371/journal.pone.0192594

**Published:** 2018-02-14

**Authors:** Ernest Baskin

**Affiliations:** Department of Food Marketing, Haub School of Business, Saint Joseph’s University, Philadelphia, Pennsylvania, United States of America; Laboratoire National de Santé, LUXEMBOURG

## Abstract

This article tests low cost interventions to increase influenza vaccination rates. By changing an email announcement sent out to employees in 2014 (n > 30,000), the following interventions are tested: incentives, attention to the negative impacts of not get vaccinated, and showing a map to the vaccination centers at the end of the email announcement. Only the map condition helped increase influenza vaccination rates. The use of low-cost interventions can improve influenza vaccination rates though not all interventions work as well as others in the field. In particular, while including maps helped increase vaccination rates, other factors such as negative impact reminders and incentives, which previous studies have found to be successful in the laboratory, did not.

## Introduction

Vaccinations are an important preventative measure for disease occurrence. In particular, they can provide protection against the influenza virus [1]. Symptoms of influenza include fever, cough, headache, sore throat, amongst others that can last from 3–7 days but can persist for weeks [2, 3], resulting in missed work and school [4]. These symptoms can also lead to the worsening of chronic diseases that could potentially lead to hospitalization and even death [3]. Due to the highly contagious nature of the influenza virus, vaccination is recommended and is the most effective preventative method [5] (recommended yearly by the Centers of Disease Control and Prevention for most Americans [6]).

These recommendations are often not heeded. According to the CDC National Immunization survey, only about 43.6% of adults and 59.3% of children get immunized every year in the United States as of 2015 [7]. There are a variety of reasons why people do not get the vaccine including worry about receiving influenza from the vaccine [8], lack of understanding of their own risk factors [9–11] or potential consequences of getting influenza [12]. Even if people understand the necessity for vaccination, many do not follow through on their intentions to get vaccinated [13].

Research has looked at ways to increase vaccinations in the healthcare setting [6, 13, 14]. These interventions can include education, defaulting people to influenza vaccination appointments [15], increasing vaccine access in health care offices [6], financial incentives [16], peer feedback and email reminders [17], amongst others. Many of these options require substantial resources on the part of the healthcare provider or the insurance carrier in order to be effective. Factors that increase influenza vaccination rates but also increase costs include increasing access to more influenza vaccination centers [18, 19] or creating various types of educational and/or motivational programs [20, 21]. This has led to new streams of research focusing on ways to increase healthy behaviors using low cost interventions such as establishing convenience salience [22, 23], altering risk perceptions [22], manipulating low cost incentives [24], etc. Much of the literature, however, did not look at influenza specifically and many of the studies were mainly done in the laboratory rather than in real-world clinical settings (with a few notable exceptions: [13, 15, 17, 22, 23]). Thus, this research builds on these findings by combining a number of well-known findings in the literature and testing to see which ones work best in a clinical setting. Notably, the research is limited to findings that can be implemented cheaply by the health care provider by embedding the treatments in an email communication being sent out to the population of interest.

The following research builds on prior findings to understand their relative strength in an email-based influenza vaccination campaign. First, prior research has suggested that increasing the salience of the availability and convenience of vaccination can increase tetanus vaccinations [22]. In addition, a study conducted at the University of Michigan suggests that implementing various forms of reminders for both patients and physicians thus increasing the salience of vaccinations during an appointment (during which it is convenient to obtain one) can increase influenza vaccinations [23]. Thus, this research tests another way of increasing convenience salience, via including a map at the end of the clinic announcement email. This showed people that the clinics were located close to their primary place of work or study. Notably, any person receiving the email could easily click through the link in the email to see the website and the list of clinics even if they were not in the map condition. Thus, this intervention did not give any additional information to the email’s readers but only increased the salience of a subset of that information. In particular, moving this information to the bottom of the email rather than requiring people to click through can address the convenience barrier to vaccination by putting the convenience of the vaccination clinics in a more salient position and not requiring readers of the email to perform an extra task. Thus, it will likely improve vaccination rates (H1). Still, most people in the map condition likely still clicked through to the link in the email as the map had two key downsides. It did not show the times that the vaccination clinics would be taking place and it did not show the actual addresses of the vaccine clinic locations but, instead, depicted the locations as stars on the campus map.

Second, previous research has shown that people’s anticipated fear, worry, and other negative emotions can influence their behavior regarding health practices such as receiving influenza vaccinations [8, 25]. In particular, fear arousal can increase tetanus vaccination rates [22]. This is consistent with regulatory focus theory which suggests that fear is generally associated with preventative actions [26]. Therefore, H2 suggests that making people fearful of the consequences of not getting a vaccination might increase influenza vaccination rates. While these consequences can vary in terms of severity and length [3], two specific consequences that seemed most relevant for our population were highlighted. One consequence was related to one’s personal life in terms of sickness while another was related to one’s professional life in terms of missed time from school/work. They were implemented as two separate conditions in order to measure whether consequence saliency, implemented in one of two ways, had differential effects on vaccination rates.

Finally, literature in economics, healthcare and psychology has suggested that people respond to incentives [27, 28]. In particular, an incentive, even a lottery based one, can increase response rates on surveys [29] and enhance weight loss [30] amongst other factors. As incentives can often act as a motivational force, H3 suggests that giving out a lottery based incentive to those getting vaccinated might increase vaccination rates. The use of a lottery rather than a flat incentive for each participant is consistent with keeping the intervention cost low.

## Methods

### Study sample

This study was approved by the institutional review board of the university described herein for a protocol that waived participant informed consent. All participants in this study (n = 30,748) were students, faculty and staff at a large university in the United States during the 2014–2015 influenza season. About 18% of the population consisted of undergraduate students between the ages of 18 and 21 while another 23% were graduate and professional students likely to be under 30. The rest of the sample is composed of various employees of the university who vary from just having received their high school degree to being close to retirement. This university offers free influenza vaccinations to all students, faculty and staff (our participants) every fall semester. The influenza vaccination program began in September with an email sent out to all members of the university at the end of September to announce the dates, times, and locations of the vaccination clinics. The email requires clicking on a link in the body of the email to see this information. This information is also available on the university health plan website and the university follows up with mailers and other messages to certain targeted segments of this population based on age and other risk factors. The clinics are held in multiple locations and during multiple times throughout the months of October and November on the university campus so as to give members of the community a variety of convenient options through which they can obtain a vaccination. These clinics are operated on a walk-in basis and do not require any appointments. On average, wait times for a vaccination at these clinics averaged about 0–10 minutes. While not all members of the community are on the university health plan, the influenza vaccination clinics are open to all faculty, staff and students in the university community, regardless of their membership status in the university health plan. As the university health plan acts as an HMO, vaccinations outside of the university are not covered as benefits for those on the university health plan. Therefore, they would be paid completely out of pocket. In addition, any vaccinations obtained through a PCP appointment rather than through a vaccination clinic are recorded in the data set as having obtained a vaccine so the vaccination of these community members is observed. Additionally, messaging regarding the availability of the clinics was also sent to all members of the community even if they did not have health insurance through the university. All members of the university community who received the email were considered to be part of the sample though they were unaware that they were participating in an experiment.

### Measures

In this study, the initial email sent out to the university population was modified. The general email that is usually sent out, and which was used as the control condition, can be seen in S1 Appendix with university name blinded. Since this email is sent out every year, participants were likely unaware that they were part of an experiment but, again, all recipients were considered to be part of the experiment with no explicit recruitment. All university members were used as participants in a study and there was no selection for race or gender. The emails were divided into 12 conditions in a 2 (Map Inclusion: Yes or No) x 3 (Negative Impacts of Reminder: None or sickness reminder or work reminder) x 2 (Incentive Inclusion: Yes or No) design. The dependent variable was whether or not the person receiving the email subsequently went and obtained a vaccination. While there was a linkage for each person receiving a vaccination back to the email that they received, no data on their employment/student status, race, or gender was received. However, due to randomization, gender and employment/student status were likely, on average, to be approximately evenly distributed across conditions. Also, no information regarding people who were decided to obtain a vaccination outside of the university health system and paid out of pocket or used a different insurance plan was obtained. However, the number of vaccinations that were not included in our sample was likely small. Randomization was conducted using an Excel randomizer function to assign each person at the university to a specific email type while emails were mailed out by the university. The author was not able match participants to emails.

In the map condition, half of the participants were given a map of campus with red stars indicating the locations of the influenza vaccination clinics. Again, this was not additional information as all participants were able to click through the link in the email to see the list of the influenza vaccination clinics. In fact, the link brought them to a more detailed list with dates and hours which the map did not contain. The map appeared at the bottom of the email. While the map was salient, it did require participants to scroll to the bottom of the email in order to view it similar to the other interventions.

In the negative impacts of influenza reminder conditions, a single sentence appeared near the end of the email. Right after the link to the university website, some participants saw “Please consider how sick you may feel if you wind up getting the flu,” while others saw “Please consider how much time you will miss from work/school if you wind up getting the flu,” while still others saw no additional sentence, which functioned as a control condition.

In the incentive condition, half of the participants were given an addition final sentence at the end of the email which said “In addition, for getting a flu vaccine, we are offering you an entry into a raffle for one of 3 Amazon.com gift certificates worth $100 if you get a flu vaccination.” The other half of the participants did not view this sentence. A post-test conducted on the undergraduate student population (a sample size of 50 students) at this university the following year (2016) showed students the email that was sent out and then asked “What is the chance that you will receive a gift certificate if you go and get a flu shot?” This survey was distributed as part of a survey packet of unrelated studies for which students were paid $10 dollars in order to participate. Students believed that, on average, they would have about a 5% chance of receiving the gift certificate if they went to get an influenza vaccine. This is likely due to not knowing the size of the university community as well as an incorrect assumption about how many people would likely get vaccinated. This suggests that people did think that they were at least somewhat likely to receive the gift certificate.

### Statistical methods

A binary logistic regression (with and without interaction terms) was conducted using influenza vaccination as the dependent variable (see Table 1 for B coefficients and significance levels). Data was manually entered by the health system after the influenza vaccination clinics and a final data pull of influenza vaccinations matched with original email message conditions was obtained after April 2015 from the health system. Analyses were conducted in 2016–2017 after study completion.

**Table 1:** All conditions and all means for study conducted in 2014–2015.

Condition	Map	Ending of email change	Lottery	% Vaccinated	Total N
1	Not Included	No change	No Lottery	32.5%	2,647
2	Included	No change	No Lottery	35.1%	2,532
3	Not Included	Please consider how sick you may feel if you wind up getting the flu	No Lottery	33.2%	2,606
4	Included	Please consider how sick you may feel if you wind up getting the flu	No Lottery	33.2%	2,521
5	Not Included	Please consider how much time you will miss from work/school if you wind up getting the flu	No Lottery	31.6%	2,462
6	Included	Please consider how much time you will miss from work/school if you wind up getting the flu	No Lottery	33.5%	2,495
7	Not Included	No change	Lottery	31.0%	2,586
8	Included	No change	Lottery	34.1%	2,605
9	Not Included	Please consider how sick you may feel if you wind up getting the flu	Lottery	31.5%	2,504
10	Included	Please consider how sick you may feel if you wind up getting the flu	Lottery	34.1%	2,595
11	Not Included	Please consider how much time you will miss from work/school if you wind up getting the flu	Lottery	32.4%	2,590
12	Included	Please consider how much time you will miss from work/school if you wind up getting the flu	Lottery	33.7%	2,605

## Results

Both models with and without interaction terms are reported in Table 2. All conditions as well as the percentage of participants who received an influenza vaccination in each condition can be viewed in Table 1. The full control condition revealed that 32.5% of the university community obtained the vaccination, which is consistent with data from prior years in terms of 30% to 33% vaccination participation. This is also consistent with CDC vaccination projections for the predominant age group in this sample. In both regressions, the only significant variable was the map condition indicating that providing a map increased the probability of getting vaccinated overall by about 2% resulting in approximately 600 additional vaccinations across the university.

**Table 2:** Logistic regression results for study conducted in 2014–2015.

	B (SE)	Sig	B (SE)	Sig
Map Included	.088 (.024)	<.01	.115 (.059)	.049
Sick Time	-.007 (.030)	.808	.030 (.059)	.608
Work Time	-.018 (.030)	.542	-.044 (.060)	.459
Lottery	-.018 (.024)	.448	-.072 (.059)	.228
Map x Sick Time			-.113 (.084)	.175
Map x Work			-.027 (.084)	.752
Map x Lottery			.028 (.083)	.736
Sick x Lottery			-.005 (.084)	.950
Work x Lottery			.108 (.085)	.201
Map x Sick x Lottery			.087 (.119)	.461
Map x Work x Lottery			-.058 (.119)	.628
Constant	-.735 (.027)	<.01	0.482	<.01

## Discussion

The results suggest that, given an average employee sick rate of 20% without the vaccine and an average cost of $1000 per employee away from work due to influenza just in lost labor hours according to the Society for Human Resource Management, adding a simple map to the correspondence related to flu vaccination clinics could potentially save the university $120,000 per year [31]. This does not include any lost revenue from a job not being performed optimally or any costs related to potential comorbidities or physician visits related to influenza. Aside from cost savings, increasing vaccination rates can lead improved community health through the development of herd immunity and may increase general awareness of influenza’s impacts. The results are likely generalizable outside the influenza vaccination context to other public health initiatives that require community members to attend a specific location including STD screenings, blood donation drives, or retailers offering vaccinations to their consumers. However, when generalizing, it is important to consider differences between the community members in this data set and people that the results may be generalized to. The results shown here may have interactions with SES and education level as well as other variables and these variables may differ, on average, in other populations. The closest level of generalizability is likely to be other workplace settings. There may also be other community-wide interventions in the population under study that are unobservable though random sampling should diminish its effects on the results.

Overall, this research suggests that making convenience salient through a map’s inclusion increased vaccination rates. There was also no requirement on the part of the healthcare plan to spend money on a mailing or the necessity for people to explicitly write down their intention to get a vaccination. Thus, the data supports H1. Notably, our intervention did not make the vaccinations themselves more convenient but increased perceptions of their convenience by making the convenience information more salient to the email reader. However, neither incentives nor reminders of the downsides of influenza were enough to increase vaccination rates (H2/H3 were not supported). This contributes to the literature on choice architecture [32] by offering a simple intervention that health systems and health insurance providers can implement in their email messaging to improve vaccination rates. It also contributes to the call for further research into ways in which influenza vaccination uptake can be increased in various areas [33].

While the methods used in this study were in the form of an email intervention, there may be other methods of increasing vaccination uptake. In particular, overt messaging has been proven effective to get people to make healthier decisions in taking the stairs over using the escalator [34, 35]. Thus, a campaign with messaging in prominent places on campus can help increase vaccination rates. This is consistent with prior research which has found that overt influenza vaccine messaging for both doctors and patients can help increase vaccination uptake [23]. In addition, to the extent that certain individuals do not pay attention to their email or do not check their email often as a function of their job, emails may not be a salient means of communication for some portion of the population. Thus, while more expensive, it may be prudent to also run a mail campaign to further make the vaccination clinics salient. Text messaging may also be used to increase salience even further though this generally requires opt-in permission of all university members and is likely not feasible to achieve for non-emergency communication. Another option shown to improve vaccination rates is having people write down a time during which they plan to go to the clinic [13]. This can be done using either mail or email. In addition, research has suggested that reimagining vaccination as an opt-in rather than as an opt-out system and thus making it inconvenient to not get a vaccination can improve vaccination rates [36]. This would involve requiring people to submit some sort of opt-out form in order to not get a vaccination.

Next, by pitting several psychological theories against each other, this research helps to understand which interventions suggested by these theories work better than others. As such, both the research looking at putting people into a prevention mindset by letting them know about the negative ramifications of not making a certain decision [37] as well as the literature suggesting the positive effects of incentives [28] would predict that both our negative ramification and incentive conditions should increase vaccination rates. However, they did not. Only the map condition did.

There are, however, several limitations in this research. First of all, results were tracked by looking at all students, faculty and staff who obtained a vaccination at the University Health Center, either through a PCP at the center or through a clinic, after the influenza vaccination season was over. All students, faculty and staff not on that list were recorded as not receiving an influenza vaccination. Thus, the data does not allow us to track which members of the university community had left prior to the influenza vaccination and thus could not be counted. No people that obtained their influenza vaccination outside of the university system were observed. However, these numbers are minimal since the university community does not experience a lot of churn in the middle of a semester and vaccinations outside the university health system are not covered by the university insurance plan. Also, plans that that do not restrict community members to the doctors at the university health plan are not offered to students. Even then, employees, who have a choice of plans, often find themselves selecting the HMO based university health plan option rather than a more traditional PPO option due to the large university premium subsidies and lack of copayments on office visits/procedures on the university health plan.

In addition, due to the nature of the data, it was not possible to include individual variables in the analysis. Thus, the research does not differentiate between the impact of the various vaccination interventions on different subgroups based on race, employment, income, gender, medical comorbidities, etc and only reports the average vaccination rates across all groups. This may be important for future research as employees might care more about missing days of work than students care about missing days of school. Also, those employed in high compensation positions in the university may feel that the incentive offered was too small relative to their salary and thus may not be as motivated by it though these staff are still randomized by condition and likely constitute a small portion of the sample.

## Supporting information

S1 Appendix Email text. (DOCX)
