# Supplementary material for: Increasing influenza vaccination rates via low cost messaging interventions
Source: PLoS One. 2018 Feb 14;13(2):e0192594. doi: 10.1371/journal.pone.0192594 (PMC5812620; doi:10.1371/journal.pone.0192594)
Supplement: S1 Appendix — (DOCX) [file pone.0192594.s001.docx]

Appendix 1: Control Email and Modifications

As I'm sure you know, flu (influenza) season is now approaching. “University Health is committed to keeping the University community “flu free" -  so each year we organize vaccination clinics where we immunize members of the University's faculty, staff and student populations at no charge.

The CDC (Centers for Disease Control and Prevention) provides valuable information about the influenza vaccine and I suggest that you look at the following web site for any questions you may have about the influenza vaccine: http://www.cdc.gov/flu/protect/vaccine/index.htm. 

Please visit our own web site (University website) to find a vaccination clinic that will work for you. I invite you to take advantage of this important University Health service as we strive to sustain a Healthy University.

The above is Condition 1. See below for the last paragraph of other conditions. The map conditions do not show the map as this would reveal the university where the study was conducted.

Condition 2

Please visit our own web site (University website) and see the enclosed map to find a vaccination clinic that will work for you. I invite you to take advantage of this important University Health service as we strive to sustain a Healthy University.

Condition 3

Please visit our own web site (University website) to find a vaccination clinic that will work for you. Please consider how sick you will feel if you wind up getting the getting the flu. I invite you to take advantage of this important University Health service as we strive to sustain a Healthy University.

Condition 4

Please visit our own web site (University website) and see the enclosed map to find a vaccination clinic that will work for you. Please consider how sick you will feel if you wind up getting the getting the flu. I invite you to take advantage of this important University Health service as we strive to sustain a Healthy University

Condition 5

Please visit our own web site (University website) to find a vaccination clinic that will work for you. Please consider how much time you will miss from work/school if you wind up getting the flu. I invite you to take advantage of this important University Health service as we strive to sustain a Healthy University.

Condition 6

Please visit our own web site (University website) and see the enclosed map to find a vaccination clinic that will work for you. Please consider how much time you will miss from work/school if you wind up getting the flu. I invite you to take advantage of this important University Health service as we strive to sustain a Healthy University

Condition 7

Please visit our own web site (University website) to find a vaccination clinic that will work for you. I invite you to take advantage of this important University Health service as we strive to sustain a Healthy University. In addition, for getting a flu vaccine, we are offering you an entry into a raffle for a gift certificate to the University Bookstore.

Condition 8

Please visit our own web site (University website) and see the enclosed map to find a vaccination clinic that will work for you. I invite you to take advantage of this important University Health service as we strive to sustain a Healthy University. In addition, for getting a flu vaccine, we are offering you an entry into a raffle for a gift certificate to the University Bookstore.

Condition 9

Please visit our own web site (University website) to find a vaccination clinic that will work for you. Please consider how sick you will feel if you wind up getting the getting the flu. I invite you to take advantage of this important University Health service as we strive to sustain a Healthy University. In addition, for getting a flu vaccine, we are offering you an entry into a raffle for a gift certificate to the University Bookstore.

Condition 10

Please visit our own web site (University website) and see the enclosed map to find a vaccination clinic that will work for you. Please consider how sick you will feel if you wind up getting the getting the flu. I invite you to take advantage of this important University Health service as we strive to sustain a Healthy University. In addition, for getting a flu vaccine, we are offering you an entry into a raffle for a gift certificate to the University Bookstore.

Condition 11

Please visit our own web site (University website) to find a vaccination clinic that will work for you. Please consider how much time you will miss from work/school if you wind up getting the flu. I invite you to take advantage of this important University Health service as we strive to sustain a Healthy University In addition, for getting a flu vaccine, we are offering you an entry into a raffle for a gift certificate to the University Bookstore.

Condition 12

Please visit our own web site (University website) and see the enclosed map to find a vaccination clinic that will work for you. Please consider how much time you will miss from work/school if you wind up getting the flu. I invite you to take advantage of this important University Health service as we strive to sustain a Healthy University. In addition, for getting a flu vaccine, we are offering you an entry into a raffle for a gift certificate to the University Bookstore.
